# Molecular detection of human papillomavirus (HPV) in highly fragmented DNA from cervical cancer biopsies using double-nested PCR

**DOI:** 10.1016/j.mex.2018.05.018

**Published:** 2018-05-31

**Authors:** Leabaneng Tawe, Surbhi Grover, Mohan Narasimhamurthy, Sikhulile Moyo, Simani Gaseitsiwe, Ishmael Kasvosve, Giacomo M. Paganotti

**Affiliations:** a Department of Medical Laboratory Sciences, Faculty of Health Sciences, University of Botswana, Gaborone, Botswana; b Botswana-Harvard AIDS Institute Partnership, Gaborone, Botswana; c Botswana-University of Pennsylvania Partnership, Gaborone, Botswana; d Department of Radiation Oncology, Perelman School of Medicine, University of Pennsylvania, Philadelphia, USA; e Department of Pathology, Faculty of Medicine, University of Botswana, Gaborone, Botswana; f Department of Immunology and Infectious Diseases, Harvard School of Public Health, Boston, MA, USA; g Department of Medicine, Perelman School of Medicine, University of Pennsylvania, Philadelphia, PA, USA; h Department of Biomedical Sciences, Faculty of Medicine, University of Botswana, Gaborone, Botswana

**Keywords:** Double-nested PCR for detecting and genotyping HPV, HPV, FFPE, DNA, Double-nested PCR, RFLP, DNA sequencing

## Abstract

Archived Formalin-Fixed Paraffin-Embedded (FFPE) tissue specimens can be a valuable source of human papillomavirus (HPV) nucleic acids for molecular biological analyses in retrospective studies. Although successful amplification with polymerase chain reaction (PCR) is essential for analysis of HPV DNA extracted from cervical FFPE specimens, extensive DNA damage due to cross-linking and fragmentation results in poor yield. Therefore, techniques to improve the diagnostic rate and sensitivity from FFPE tissues through PCR is highly desired and of wider interest. To overcome this, a highly sensitive double-nested PCR methodology was designed and optimized for limited-resource laboratories coupled with an organic extraction of DNA. This method allows the detection of a broad range of HPV genotypes and also allowing the sequencing of the final amplicon. Validation of the new approach developed was done with an automated DNA extraction coupled with Real Time PCR. Results showed that the proposed method achieves 96.3% of HPV detection as compared to 100% Abbott m2000rt used as ‘gold standard’. Moreover, the concordance rate between the two methods was equal for detecting HPV -*16* or -*18* genotypes. Nevertheless, the newly introduced assay has an advantage of:

• Simultaneously identifying broad range of HPV genotypes besides HPV-*16* and -*18* from clinical samples.
• It is an easy and cost-effective method that can be beneficial in resource-limited setting and can be employed for various molecular applications.
• The method is indicated for highly degraded FFPE samples.

## Graphical abstract

**Figure:**
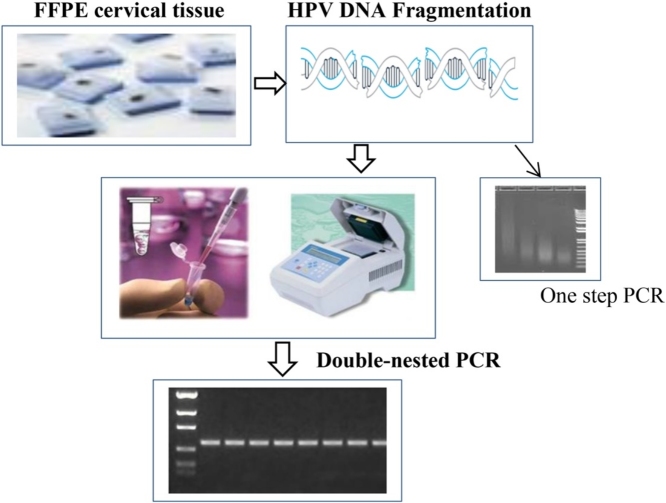

Specifications Table

**Table:** 

Subject area	Biochemistry, Genetics and Molecular Biology
More specific subject area	Molecular detection and genotyping
Method name	Double-nested PCR for detecting and genotyping HPV
Name and reference of original method	J. Coser, T.R. Boeira, A.S. Fonseca, N. Ikuta, V.R. Lunge, Human papillomavirus detection and typing using a nested-PCR-RFLP assay, Braz. J. Infect. Dis. 15 (2011) 467–72. L. Chen, K. Watanabe, T. Haruyama, N. Kobayashi, Simple and rapid human papillomavirus genotyping method by restriction fragment length polymorphism analysis with two restriction enzymes, J. Med. Virol. 85 (2013) 1229–34.

## Introduction

Human papillomavirus (HPV) has been associated with most cervical cancers in women [1]. More than 100 genotypes of HPV have been identified to date in several human tissues [2]. However, only 40 genotypes are known to infect the genital tract [2] and 12 genotypes are known to be oncogenic [3,4]. It is of clinical and public health importance to detect and genotype HPV in a sensitive and specific manner to characterize HPV. This is of utmost importance in low- and middle-income countries (LMICs) and in particular in Africa since there is limited data on HPV genotypes found in HPV-associated cancers. In LMICs diagnosis of HPV infection is often limited to consider the presence of pre-cancerous and cancerous processes in the cervix [5]. HPV testing is mainly performed using dry swabs, liquid-based cytologies, but also formalin-fixed paraffin-embedded (FFPE) biopsies. The FFPE specimens are taken from selected target regions of the cervical epithelium, allowing detection of HPV genotypes through DNA analysis. The genotypes can then be linked to specific lesions.

**Fig. 1:**
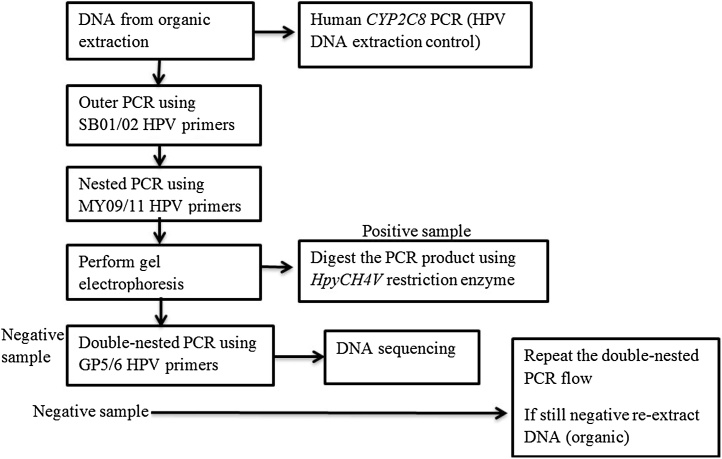
Workflow diagram for HPV detection from FFPE samples.

Molecular methods to detect HPV-DNA (mainly the oncogenic genotypes) have already been developed [6] and are currently used in clinical laboratories worldwide, but rarely applied in Africa. HPV molecular testing is challenging in FFPE samples due to poor DNA quality in the embedded tissue [[7], [8], [9], [10], [11]], resulting in lower HPV detection rate and impairing the identification of multiple infections in comparison to fresh cervical smears. Consequently, it has also been reported that successful amplification of HPV sequences from archived FFPE specimens is inversely correlated to the length of the PCR amplicon and that specimen age may contribute to degradation [7,12,13]. Importantly, the advantage of FFPE samples is that they do not require specific storage conditions and they can be preserved at room temperature for long time and therefore avoids the complexity and risk of freezing. Genotyping methods for HPV vary by target sequence and amplicon size. Standard genotyping methods often cannot be easily applied to FFPE specimens. Particularly, formalin fixation may cause extensive DNA damage, including cross-linking and fragmentation [12,[14], [15], [16], [17], [18]]. Several techniques for the molecular diagnosis of HPV, ranging from conventional PCR methods, Real Time PCR, to hybrid capture and microarrays has been proposed [6,[19], [20], [21]]. The PCR technique is still considered the “gold standard” for HPV diagnosis, as the DNA target is selectively amplified. A variation of this technique, a nested PCR with the MY09/11 and GP5+/6+ primer sets, is a high sensitive specific method for HPV DNA detection [22] with both targeting the L1 conserved region of the viral genome (Gene ID: 1489082), allowing the detection of a broad range of HPV genotypes. However, the majority of the PCR methods use single PCR amplification with two pairs of consensus primers: MY09/11 or GP5/6 or GP5+/6+ [[23], [24], [25]]. Because FFPE samples are usually highly degraded, in the extracted DNA template the concentration of relatively long fragments isolated is low and cannot be amplified successfully by standard procedures. To achieve the goal of typing HPV from highly fragmented DNA samples, we developed a method based on adapting previously existing protocols in a new workflow (Fig. 1).

The present approach is based on the three subsequent amplifications by PCR: an outer PCR (with SB01/02 primers), a nested PCR (with MY09/11 primers) and double-nested PCR (with GP5/6 primers) with several modifications. Moreover, with high-degraded DNA templates, human beta-globin gene used as a DNA extraction control could not be amplified successfully, therefore an in-house validated diagnostic PCR for human *CYP2C8* (Cytochrome P450 Family 2 Subfamily C Member 8) gene was used [26] and adopted as DNA extraction control (Gene ID: 1558). The advantage is due to a short PCR amplicon (107 bp) of the *CYP2C8* compared with beta-globin gene (268 bp). Given the importance of accurately identifying HPV present in FFPE materials, the suggested double-nested PCR method was compared with the HPV detection rate through the Abbott Real Time High risk (HR) (cancer HR genotypes) HPV assay [27].

### Ethics statement

Ethical approval was obtained from the Institutional Review Board (IRB) at the University of Botswana (Protocol # UBR/RES/IRB/1636), Ministry of Health and Wellness, Botswana (Protocol # HPDME: 13/18/1 Vol. × 324) and University of Pennsylvania's IRB (Protocol # 820159). The IRB gave an exemption for written informed consent from the participants.

### Study population and sample collection

One hundred thirty four (134) cervical cancer biopsy samples collected and stored by the National Health Laboratory (NHL) in Gaborone, Botswana, between 2013–2015 were used for this retrospective study. The Anatomical Pathologist at the Botswana’s Ministry of Health and Wellness NHL made cervical cancer diagnosis. NHL is one of two national laboratories where all clinical samples from the public hospitals in Botswana are processed and stored. The samples were selected following the guidelines of the Helsinki Declaration of 2000.

## Methods

### Tissue sectioning

From each tissue block, 10 μm thick sections were cut and placed individually in a 15 ml tube and sent to the molecular laboratory for HPV genotypes detection. We followed strict aseptic techniques when handling tissue blocks and avoided using one microtome blade for different blocks to minimize cross contamination. To check for potential cross contamination, blank paraffin control blocks without any tissue were cut after every 10^th^ sample and analyzed for HPV DNA presence.

### DNA extraction

An organic DNA extraction was performed following the Genomic Medicine Biorepository protocol [28] with some modifications. Dewaxing was performed in 15 ml tubes using xylene for 18 h, then samples were immersed in 100% alcohol for further 18 h and lastly in a descending alcohol concentration (95%, 80%, 70% and 50%). Gradual re-hydration of the tissue sections was done by subsequent one-hour incubations in each alcohol concentration for three times.

### DNA extraction control

**Fig. 2:**
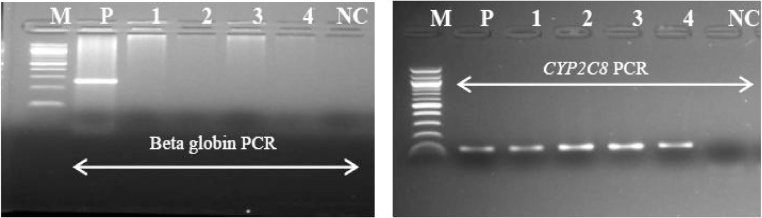
Comparative amplification of a DNA template from FFPE tissues using beta-globin and *CYP2C8* primer pair. M: molecular marker (100 bp), NC: negative control, P: positive control. Agarose gel (2%) showing PCR amplified DNA fragments of different sizes within beta-globin (268 bp) on the left gel and *CYP2C8* (107 bp) on the right gel gene locus using template from DNA from fragmented FFPE tissue. As seen, clearly PCRs was unsuccessful using beta-globin primer pair compared to *CYP2C8* primers.

Human beta-globin gene is normally used to assess whether adequate cellular DNA has been extracted. All the samples were first analysed using beta-globin primers (Fig. 2). To overcome poor yield due to high DNA fragmentation (also in human tissues), we decided to amplify a relatively short portion of the human *CYP2C8* gene as a DNA extraction control, obtaining a 107 bp amplicon (Fig. 2) [26]. Forward and reverse primers are shown in Table 1. Human DNA was used as a control for each of the extracted samples.

**Table 1:** Oligonucleotide specifications and amplicon sizes.

Name of the primer	Sequence (5′–3′)	Amplicon size and Reference
aSB01	CAAWTRTTYAATAARCCWTATTGG	∼495 bp [29]
aSB02	AAAAAYTTYCGWCCMARRGG	

aMY09	CGTCCMARRGGAWACTGATC	∼450 bp [31]
aMY11	GCMCAGGGWCATAAYAATGG	

GP5	TTTGTTACTGTGGTAGATAC	∼150 bp [32]
GP6	GAAAAATAAACTGTAAATCA	

CYP2C8-F	GAACACCAAGCATCACTGGA	107 bp [26]
CYP2C8-R	GAAATCAAAATACTGATCTGTTGC	

a Primers are degenerated.

### Conventional PCR analysis

#### Outer PCR analysis

HPV DNA samples were analyzed by first applying SB01/02 PCR primers (Table 1) according to Coser et al. [29] To increase the HPV detection rate the assay was modified and optimized for template concentration (3 μl in a final volume of 20 μl reaction), annealing temperature (42 °C), duration of each step (as indicated in Table 2), number of cycles (from 20 to 40 in two steps), and the MgCl_2_ concentration that was adjusted to 3.5 mM. Thermal cycling conditions for outer PCR (Table 2) were modified from Coser et al. [29]. PCR product was approximately 495 bp for the majority of HPV genotypes. Positive and negative controls were included with all PCR series and were analyzed in parallel with the clinical specimens in the subsequent nested PCR analysis.

**Table 2:** Modified PCR cycling for HPV detection analysis from FFPE tissues.

Process	Temperature (°C)	Duration	Number of cycles
**SB01/SB02 primers**			
Pre-denaturation	95	5min	1
Denaturation	95	1min	10
Annealing	42	1min	
Extension	72	1min	
Denaturation	95	20s	30
Annealing	42	40s	
Extension	72	1min	
Final extension	72	10min	1

**MY09/11 primers**			
Pre-denaturation	95	5min	1
Denaturation	95	15s	40
Annealing	50	1min	
Extension	72	1min	
Final extension	72	10min	1

**GP5/6 primers**			
Pre-denaturation	95	10min	1
Denaturation	95	30s	40
Annealing	50	40s	
Extension	72	30s	
Final extension	72	10min	1

#### Nested PCR analysis

**Fig. 3:**
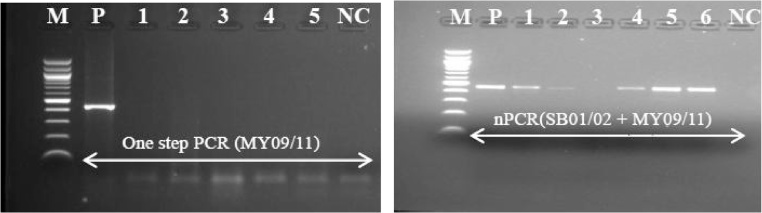
Comparative amplification of HPV DNA template using one step PCR (MY09/11) protocol and nested PCR (MY09/11) from FFPE tissues. M: molecular marker (100 bp); P: positive control; NC: negative control. Numbers refer to samples. Both nested PCR carried out using 3 μl template DNA and PCR amplicons were run on 2.5% agarose gel stained with ethidium bromide. Nested PCR produce better yield of PCR product compared to one step PCR.

**Fig. 4:**
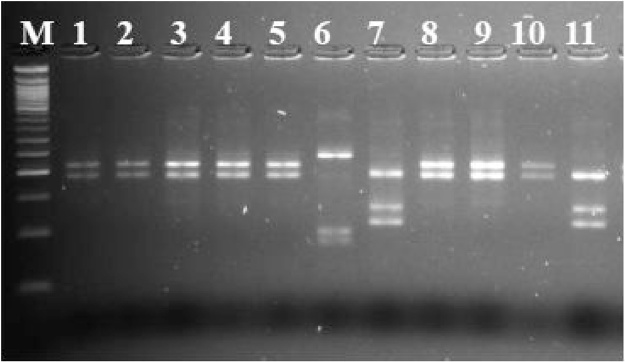
RFLP digestion pattern of nested PCR. The digested PCR products from nested PCR (SB01/02 + MY09/11) (line 1–11) and DNA molecular weight (100 bp) marker (M) were electrophoretically visualised using MetaPhor^™^ agarose gel (4%) (Lonza Rockland, ME, USA) stained with ethidium bromide showing digested PCR products from nested PCR using *HpyCH4V* restriction enzyme (Line 1–5, 8–10; HPV-*16*, line 7 and 11; HPV-*33*, line 6; HPV-*45*).

Second round of PCR amplification was carried out with consensus primers MY09/11 (Table 1). MgCl_2_ concentration was adjusted to 3.5 mM. Thermal cycling conditions were adopted from Cheng et al. [30] with some modifications (Table 2); positive and negative samples were added as controls in all the experiments for quality control. Amplified reaction products underwent electrophoresis in agarose gel and positive and negative results were evaluated based on the presence of fragments of the expected size pair combination (∼450 bp) (Fig. 3). All HPV positive samples were also typed by restriction fragment length polymorphism (RFLP) analysis (Fig. 4) according to Cheng et al. [30] using *HpyCH4V* restriction enzyme. This allows to obtain genotypes information for nested PCR positive samples.

#### Double-nested PCR analysis

**Fig. 5:**
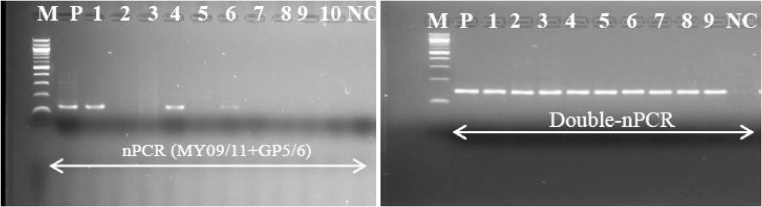
Comparative amplification of HPV DNA template using PCR MY09/11 followed by GP5/6 primers and double-nested PCR protocol from FFPE tissues. M: molecular marker (100 bp), P: positive control; NC: negative control. Numbers refer to samples. NPCR: nested PCR; double-nPCR: double-nested PCR. Both PCR were carried out using 3 μl of the DNA template and amplicons (∼150 bp) were visualized on the 2% agarose gel stained with ethidium bromide.

All the HPV negative samples from nested PCR (Fig. 3) were then subjected to another cycle of PCR amplification using GP5/6 primers’ pair (Table 1). MgCl_2_ concentration was set to 2.5 mM. The reaction was carried out in total volume of 20 μl using 3 μl of the PCR amplicons from nested PCR analysis. Modified thermal cycling conditions (Table 2) were applied. Positive and negative samples were added as controls in all the experiments for quality control. Amplified reaction products and molecular size markers were underwent electrophoresis in agarose gel (Fig. 5). Different negative controls testing the different stages in the PCR process at which contamination may occur were also included. In case contamination is detected, all the diagnostic work was stopped until source of contamination is identified and cleared.

### DNA sequencing

Since a RFLP protocol has not been developed based on the previous assay with the GP5/6 primers, we proceeded with sequencing of the PCR products to identify the HPV genotypes. Sequencing PCR was performed using the general primers GP5 and GP6, and the respective sequences of the HPV DNA regions corresponding to the two primer sets were read using the Applied Biosystems DNA analyzer. About 1 μl of the double-nested PCR purified product was used together with 1 μl of 5 μM GP5 and GP6 as the sequencing primer. Resulting DNA sequences were analyzed using the Basic Local Alignment Search Tool (BLAST) database on the website of the NCBI to confirm specific genotypes.

### Abbott HR HPV genotyping

Abbott Real Time HR HPV assay was applied to compare the proposed approach with an established high throughput method [27]. The main disadvantages are the high cost of the kits, commercial unavailability in many countries and requirement for special equipment. The Abbott platform automatically extracts and detects DNA of known 14 HR HPV genotypes using modified GP5+/6+ primer mix consisting of three forward primers and two reverse primers targeting the conserved L1 region. The assay provides specific probes that are differentially labelled qualitatively for the detection of HPV*-16* and *-18*, and an evaluation of the human beta-globin internal control [27]. All 134 cervical specimens were thus analysed through Abbott Real Time HR HPV assay for comparison and validation.

### Statistical analysis

Chi-square (X^2^) test for trend was used to evaluate if there was a statistical significant increase in the HPV detection rate due to the technique applied (one step PCR, nested PCR and double-nested PCR). The results were considered to be statistically significant with a *P* value below 0.05.

## Results

One hundred thirty four (134) HPV positive samples diagnosed through microscopy were selected for this procedure. All 134 samples were amplified for the *CYP2C8* gene in substitution of human beta-globin gene. The results of human beta-globin amplification from archival tissues showed very low amplification rate compared to 100% amplification rate of *CYP2C8* (Fig. 2). The proposed double-nested PCR method showed 96.3% (n = 129) of HPV detection rate compared with the Abbott Real Time HR HPV assay. The Abbott Real Time HR HPV assay showed 100% (n = 134) HPV positivity rate, confirming the HPV presence in cervical cancer biopsies. Table 3 shows the detection rate according to the method implemented. The two assays also produced matching results for HPV -*16* and -*18*. Results showed a statistical significant trend for increasing HPV detection rate (X^2^ = 237.07; *P* value < 0.00001) according to the approach described (one step PCR, nested PCR and double-nested PCR). Importantly, the described approach is able to provide detailed information for HR genotypes other than HPV -*16* or -*18* (Fig. 4). Finally, all the positive samples through double-nested PCR (96.3%) were sequenced using the general primers GP5 and GP6, edited using a App-V 5.0 sequencer and then compared to GenBank using Basic Local Alignment Search Tool (BLAST) database on the website of the NCBI to confirm specific genotypes.

**Table 3:** Comparison among several primer combination PCRs and Abbott m2000sp system method. In brackets are the primer pairs and their combination. nPCR refers to nested PCR.

	One step PCR (MY09/11)	nPCR (SB01/02 + MY09/11)	nPCR (MY09/11 + GP5/6)	Double-nPCR (SB01/02 + MY09/11+ GP5/6)	Abbott m2000rt system
Positive	4 (2.99%)	58 (43.3%)	74 (55.2%)	129 (96.3%)	134 (100%)
Negative	130 (97.01%)	76 (56.7%)	60 (44.8%)	5 (3.7%)	0 (0%)
Total	134	134	134	134	134

## Discussion

Extracting DNA from FFPE tissue remains a challenge, despite numerous attempts to develop a more effective method. PCR success rates with DNA extracted using current methods remain low. Several comparative studies of different methods for HPV-DNA detection have been proposed. Husnjak et al. [33] used conventional PCR with primers MY09/11 compared with nested PCR based on [MY09/11 + GP5/GP6]. Entiauspe et al. [21] reported the increase in HPV DNA detection from 6.8% to 29.9% using the technique of nested PCR, first applying MY09/11 primers followed by GP5/6 primers. Therefore in this report we evaluated cervical biopsy samples, and observed that using double-nested PCR the positivity rate of HPV DNA detection increased from 2.99% (n = 4) using one step PCR to 96.3% using double-nested PCR. Furthermore, we achieved 100% of *CYP2C8* detection rate that is comparable to the detection rate of human beta-globin through Abbott m2000rt platform. In this work *CYP2C8* false negative results were not found.

In our laboratory, we often use the present protocol to enhance and overcome poor yield due to high DNA fragmentation from FFPE tissues. All samples, irrespective of the year in which they were fixed and stored, could be amplified effectively through this protocol. Our results suggest double-nested PCR technique for HPV-DNA detection from FFPE as an alternative method to identifying women at high risk for cervical cancer development. Moreover, it emphasizes the importance of molecular diagnostic methods as a complementary tool to conventional preventive screenings. This method can also be used on archived samples in LMICs. To the best of our knowledge, this is the first report of the use of HPV double-nested PCR analysis for detection and typing. The novel method and workflow introduced here is simple, effective and requires no special equipment. We think it will be applicable for various molecular analyses of DNA samples from long-term preserved FFPE tissue specimens in general. In addition, PCR using DNA from FFPE tissue is often inhibited which can be alleviated by adaptation of the PCR cycling conditions, resulting in a significant improvement in PCR performance and ultimately, improved reaction specificity and sensitivity. We adapted the existing PCR system and optimized the protocols to facilitate the accuracy and sensitivity of HPV genotyping. The solutions that we have presented here are of tremendous value in clinical and laboratory medicine. However this method can take 3 to 5 days to produce definitive results, which is not satisfactory for allowing rapid product release. Therefore, it is of economic interest to continue investigating alternative rapid methods. Processing and transferring amplicons (from two PCR steps) to a subsequent PCR increases the risk of contamination. To mitigate this, we recommend the use of three distinct work stations for the sample preparation stage, the PCR setup stage and the post-PCR stage. This recommendation applies to equipment as well. In addition, positive and negative controls should be carefully checked.

## Conclusion

The results indicate that double-nested PCR produced a significant improvement of the HPV detection rate from highly degraded FFPE specimens. We also suggest to use *CYP2C8* gene in substitution of human beta-globin gene, checking DNA quality for HPV detection by PCR. The proposed method is easy, cost-effective and can be beneficial to resource-limited settings.

## Conflict of interests

The authors declare that there are no conflicts of interest.
